# Understanding the Impact of Skull Base Osteomyelitis: A Retrospective Analysis of 14 Cases

**DOI:** 10.7759/cureus.77684

**Published:** 2025-01-19

**Authors:** Padmanabhan Karthikeyan, P.S. Divya, Kirubhagaran Ravichandran, Venkataramani Agathiyanathan

**Affiliations:** 1 Otolaryngology - Head and Neck Surgery, Mahatma Gandhi Medical College and Research Institute, Affiliated With Sri Balaji Vidyapeeth (SBV), Pondicherry, IND

**Keywords:** chronic otitis externa, diabetes, eac - external auditory canal, ent emergencies, moe - malignant otitis externa, necrotizing external otitis, neuro-otology, otitis externa, otology, skull base pathologies

## Abstract

Purpose

Skull base osteomyelitis (SBO) or malignant otitis externa (MOE) is an invasive bacterial infection (a rarely fungal as well as potentially aggressive infection that involves the external auditory canal up to the temporal bone and skull base. This study provides insight into the various clinical presentations of skull base osteomyelitis, the effectiveness of different treatment approaches, and the overall prognosis of SBO based on our case series.

Materials and methods

This observational study comprises 14 SBO cases, including their diagnosis, follow-up, and treatment.

Results

Otalgia and purulent otorrhea were observed in all our cases as common symptoms. All patients were diabetic. Three cases (21%) presented with facial palsy and two cases (14%) reported a rare progression of SBO resulting in septic arthritis of the temporomandibular joint. In our study, 43% of the cases diagnosed with SBO had resistance to ciprofloxacin. All our patients had daily cleaning of the auditory canal and the application of antimicrobial ear drops along with long-term systemic antibiotic therapy followed by three weeks of oral antibiotic therapy. All our cases were reviewed after three weeks. Resolution of the disease was achieved.

Conclusion

Early diagnosis, good control of blood glucose levels, prolonged medical management, and local debridement are likely to result in a better prognosis for patients.

## Introduction

Skull base osteomyelitis (SBO), also known as necrotizing otitis externa (NOE) or malignant otitis externa (MOE), is an invasive bacterial infection as well as potentially aggressive infection that involves the external auditory canal (EAC) up to the temporal bone and skull base. Meltzer first reported SBO in 1959 [1]. *Pseudomonas aeruginosa* is a common causative organism, reported in up to 95% of cases in some studies [2]. *Pseudomonas aeruginosa* is a ubiquitous gram-negative rod. SBO is typically seen in elderly diabetes mellitus (DM) patients or immunocompromised patients.

SBO often presents with initial symptoms, such as ear pain (otalgia), discharge from the ear (otorrhea), and a sensation of fullness in the ear (aural fullness). These symptoms overlap with those of otitis externa (OE), potentially complicating diagnosis and delaying the initiation of appropriate treatment. The distinction is made when symptoms persist or worsen despite standard OE treatment, with red flags such as severe pain, cranial nerve involvement, or systemic signs. In more severe cases of SBO, patients may develop cranial neuropathies, with facial nerve palsy occurring in 17% to 26% of instances [3].

Antipseudomonal antimicrobials are the mainstay of therapy for malignant external otitis. Prior to the development of systemic agents, the mortality from this disease approximated 50%, with frequent recurrences. The cure rate has increased to 90% with the introduction of fluoroquinolones [1].

This study provides insight into the various clinical presentations of SBO, the effectiveness of different treatment approaches, and the overall prognosis of SBO based on our case series.

## Materials and methods

Fourteen consecutive patients admitted to our hospital, Mahatma Gandhi Medical College and Research Institute (MGMCRI), affiliated with Sri Balaji Vidyapeeth (SBV), Puducherry, with a diagnosis of skull base osteomyelitis (SBO) within a period of six months from July 2023 to December 2023 were selected for this study. The diagnosis of SBO was based on Cohen and Friedman’s diagnostic criteria (Table 1) of major and minor signs. The patients' average age was 71 years (min 50 - max 87 years). The clinical presentation, management, outcome, and complication of SBO were analyzed in our case series.

**Table 1:** The diagnostic criteria for skull base osteomyelitis (SBO) The diagnostic criteria for SBO were divided into two categories: obligatory and occasional. All of the obligatory criteria must be present in order to establish the diagnosis. The presence of occasional criteria alone does not establish it [4].

Diagnostic criteria of skull base osteomyelitis
Major (obligatory) signs
1) Pain
2) Exudate
3) Edema
4) Granulations
5) Micro abscesses
6) Positive technetium-99 (99Tc) scan or failure of local treatment after more than one week
Minor (occasional) signs
7) Pseudomonas
8) Positive radiograph
9) Diabetes mellitus
10) Cranial nerve involvement
11) Debilitating conditions
12) Old age

## Results

Out of 14 patients, 12 were male and 2 were female. On clinical history, patients presented with purulent otorrhea and otalgia, which were observed in all our patients. All of our cases presented with granulation tissue and polyps in the EAC on examination. On examination, tympanic membrane perforations were present in two cases. All 14 patients had a preexisting diagnosis of diabetes mellitus for more than 10 years. All the patients had documented microbiological evidence of *Pseudomonas* in ear swab cultures. There was a left-sided predominance of disease (11 cases of left SBO), with no involvement of the contralateral side. The patients' average age was 71 years (min 50 - max 87 years). The most frequent symptoms observed initially were prolonged ear pain and discharge. Notably, the ear pain was severe and frequently occurred during nighttime (termed nocturnal otalgia). Glycated hemoglobin (HbA1c) levels were recorded in all the patients. Almost all our patients (14 cases) underwent high-resolution computed tomography (HRCT) of the temporal bone. Cases showed evidence of soft tissue density in the left EAC with involvement of the disease around the mastoid and middle ear. Four cases had evidence of bony erosion (Table 2).

**Table 2:** Finding of HRCT temporal bone scans HRCT: high-resolution computed tomography

Findings	Numbers
Involvement of disease outside EAC	14
Evidence of bone erosion	4
Intracranial involvement	-

**Figure 1:**
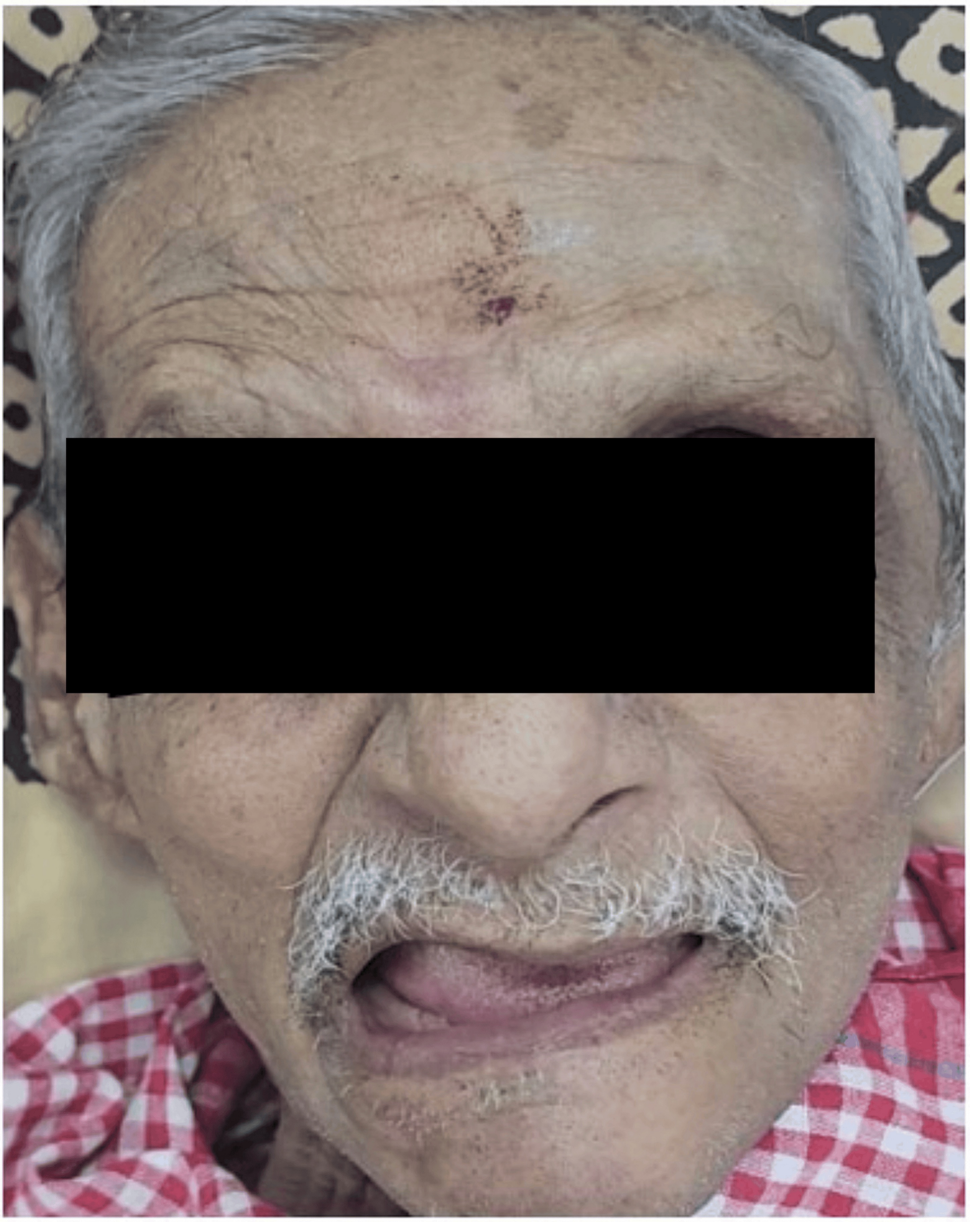
Left LMN facial palsy LMN: lower motor neuron

**Figure 2:**
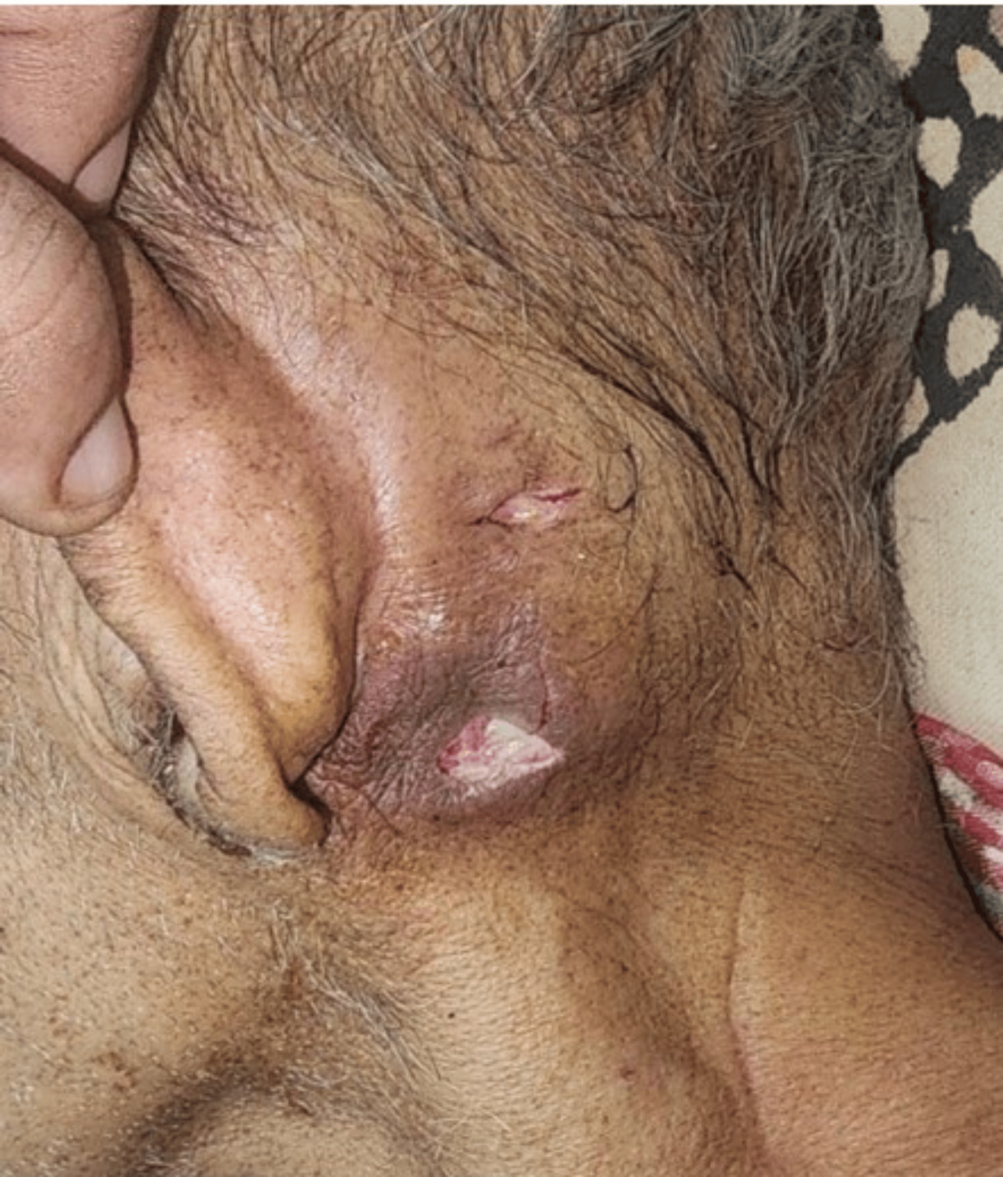
A postoperative picture of the incision and drainage done for a left subperiosteal abscess

In our series of cases, there were no fatalities associated with SBO. Out of the 14 patients, 3 (23%) had presented with seventh cranial nerve palsy (Table 3, Figure 1), and 1 patient presented with left subperiosteal abscess requiring incision and drainage (Figure 2).

**Table 3:** Summary of clinical presentations, outcomes, and management of complicated (involving the seventh nerve of the TMJ) SBO cases TMJ: temporomandibular joint; SBO: skull base osteomyelitis; EAC: external auditory canal

Cases of SBO presenting with complications						
CASE	9	10	11	12	13	14
Age/Sex	85/M	82/M	52/F	70/M	76/M	87/F
Symptoms	Left ear pain, Left ear discharge, Swelling behind the left ear.	Left ear pain	The patient came with a recurrence of left ear pain after 2 months.	The patient came with a recurrence of the disease after 3 months with left ear pain and left preauricular area swelling.	Right ear pain, swelling in front of the right ear.	Right ear pain, swelling in front of the right ear.
Ear examination	Left postauricular - A diffuse, erythematous, warm, and tender swelling of approximately 3x2 cm present; it was cystic, fluctuant, with no pus point, and no discharging sinus.			Left preauricular region - swelling +, warmth +.	Right preauricular region - swelling +, tenderness +, warmth +	Right preauricular region - swelling +, tenderness +, warmth +
Duration of diabetes (years)	35	40	10	25	20	35
On regular treatment for diabetes	+	+	+	+	+	+
Total count cells/cumm	18700	10000	8000	6400	8000	8400
HbA1c (%)	8.8	10	11	8.6	8	8
Pus culture sensitivity	Pseudomonas aeruginosa, Klebsiella pneumoniae	Pseudomonas aeruginosa, Klebsiella pneumoniae	Pseudomonas aeruginosa, Klebsiella pneumoniae	Pseudomonas aeruginosa	Pseudomonas aeruginosa	-
Cranial nerve involvement	Left lower motor neuron facial palsy (Grade II)	Left lower motor neuron facial palsy (Grade IV)	Left lower motor neuron facial palsy (Grade I)	-	-	-
HRCT	+	+ / MRI +	+	+	+ ( Figure 5)	+
Resistance to ciprofloxacin	+	+	+	+	-	-
Treatment given	Piperacillin and tazobactam 4.5g (IV,1-1-1) for 10 days. Ceftazidime 1g, IV, 1-0-1 for 5 days. Tab. levofloxacin 500 mg: 1-0-1 for 3 weeks	Piperacillin and tazobactam 4.5 g (IV, 1-1-1) for 10 days. Ceftazidime 1g IV, 1-0-1 for 5 days. Tab. levofloxacin. 500 mg: 1-0-1 for 3 weeks	Pperacillin and tazobactam 4.5g (IV, 1-1-1) for 10 days. Ceftazidime 1g: IV, 1-0-1 for 2 days 3. Tab. levofloxacin 500mg: 1-0-1 for 3 weeks	Piperacillin and tazobactam 4.5g (IV,1-1-1) for 10 days. Tab. levofloxacin 500mg: 1-0-1 for 3 weeks	Tab levofloxacin 500 mg 1-0-1 for 4 weeks	Tab levofloxacin 500 mg 1-0-1 for 4 weeks
Examination under microscopy	Granulation tissue with aural polyp excision and biopsy	Granulation tissue with aural polyp excision and biopsy	Granulation tissue with aural polyp excision and biopsy	Granulation tissue with aural polyp excision and biopsy.	-	Anterior wall of EAC granulation +
Biopsy results	Inflammatory aural polyp with dead bone	Inflammatory aural polyp with dead bone	Inflammatory aural polyp with dead bone	Inflammatory aural polyp	-	-
Surgical procedures	Incision and drainage of the left subperiosteal abscess under local anesthesia (Figure 2)	Left mastoid exploration	Left aural polypectomy with surgical debridement of EAC	Left aural polypectomy with surgical debridement of EAC	-	-
Diagnosis	Left SBO with left subperiosteal abscess, left LMN facial palsy	Left SBO with left LMN facial palsy	Left SBO with left LMN facial palsy	Left SBO with septic arthritis of the left TMJ	Right SBO with septic arthritis of the right TMJ	Right SBO with septic arthritis of the right TMJ

**Figure 3:**
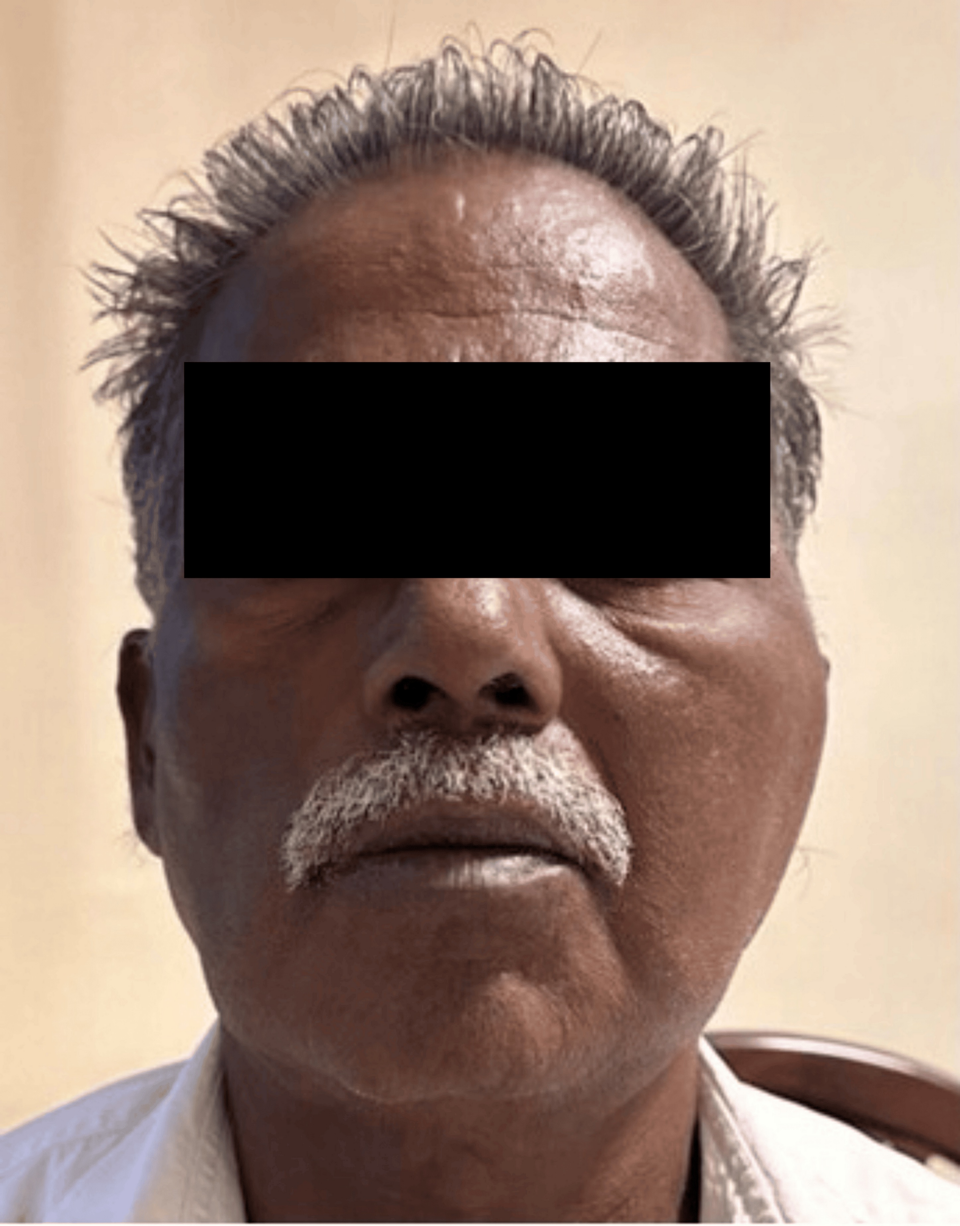
Left TMJ septic arthritis (left preauricular swelling) TMJ: temporomandibular joint

Three cases (23%) presented with a rare progression of SBO, resulting in septic arthritis of the TMJ, which was treated with oral antibiotic levofloxacin, and excision of granulation tissue was performed, which showed complete resolution (Figure 3, Table 3).

**Figure 4:**
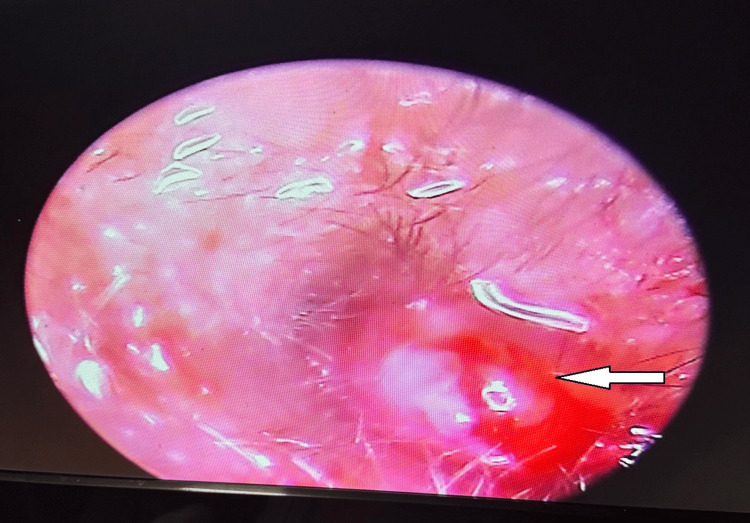
Otoendoscopy of the left external auditory canal showing left aural polyp and granulation tissue (arrow)

All our patients had daily cleaning of the auditory canal, application of antimicrobial ear drops, and long-term systemic antibiotic therapy followed by three weeks of oral antibiotic therapy. Five patients had betamethasone (0.1% w/w) plus neomycin (0.5% w/w) ointment filled in the EAC over the granulation, out of which four patients had significant improvement with reduction of granulation tissue (Figure 4).

Local debridement of granulating tissue was performed in patients. The patients were prescribed a three-week course of oral ciprofloxacin at a dosage of 500 mg twice daily for treatment. Since, in our case series, 6 (43%) cases had resistance to ciprofloxacin, these patients were initially treated with intravenous ceftazidime or intravenous piperacillin and tazobactam for 7 to10 days along with oral levofloxacin for three weeks. We noted that six cases having resistance to ciprofloxacin had an elevated risk of experiencing recurrences, advancing to facial nerve palsy, and developing septic arthritis of the TMJ. A resolution of the disease was achieved in almost all our cases. All our cases were reviewed after three weeks (Tables 3, 4).

**Table 4:** Summary of clinical presentation, outcome, and management of uncomplicated SBO cases TMJ: temporomandibular joint; SBO: skull base osteomyelitis; HRCT: high-resolution computed tomography; MOE: malignant otitis externa

Cases of SBO (Uncomplicated)								
CASE	1	2	3	4	5	6	7	8
Age/Sex	58/M	70/M	70/M	85/M	62/M	50/M	70/M	70/M
Symptoms	Left ear pain, left ear blocking sensation, left ear discharge	Left ear pain, left ear discharge, Left ear blocking sensation	Left ear pain, left ear blocking sensation	Left ear pain, left ear discharge, and left hard of hearing	Left ear pain	Left ear pain and left ear discharge	Right ear pain	Right ear pain
Duration of diabetes (years)	15	10	15	20	15	12	15	7
On regular treatment for diabetes	+	-	+	-	+	+	+	+
Total count cells/cumm	10,900	9,000	6800	8400	9200	9200	9200	
HbA1c (%)	11.2	12.2	7.3	7.1	14	9.4	14	7.5
Pus culture sensitivity	Pseudomonas aeruginosa, Klebsiella pneumoniae	Escherichia coli, Pseudomonas aeruginosa	Pseudomonas aeruginosa, Klebsiella pneumoniae	Pseudomonas aeruginosa, Staphylococcus	Pseudomonas aeruginosa, Staphylococcus	Pseudomonas aeruginosa	Pseudomonas aeruginosa, Staphylococcus	Pseudomonas aeruginosa
Cranial nerve involvement	-	-	-	-	-	-	-	-
HRCT	+	+	+	+	+	+	-	-
Resistance to ciprofloxacine	-	-	-	+	-	+	-	-
Treatment given	Ciprofloxacin 400mg (IV, 1-0-1) for 7 days. Ciprofloxacin 500mg (Per oral, 1-0-1) for 3 weeks	Ciprofloxacin 400mg (IV, 1-0-1) for 10 days. Ciprofloxacin 500mg (Per oral, 1-0-1) for 3 weeks	Ciprofloxacin 400mg (IV 1-0-1) for 7 days. Ciprofloxacin 500mg (Per oral, 1-0-1) for 3 weeks	Ceftazidime 1g (IV 1-0-1) for 5 days. Levofloxacin 500mg (Per oral, 1-0-1) for 3 weeks	Ciprofloxacin 400mg (IV 1-0-1) for 7 days. Ciprofloxacin 500mg (Per oral, 1-0-1) for 3 weeks	Piperacillin and tazobactam 4.5g (IV,1-1-1) for 10 days. Levofloxacin 500mg (Per oral, 1-0-1) for 3 weeks	Ciprofloxacin 400mg (IV, 1-0-1) for 7 days. Ciprofloxacin 500mg (Per oral, 1-0-1) for 3 weeks	Ciprofloxacin 400mg (IV, 1-0-1) for 7 days. Ciprofloxacin 500mg (Per oral, 1-0-1) for 3 weeks
Examination under microscopy	Granulation tissue with aural polyp excision and biopsy	Granulation tissue excision and biopsy	Granulation tissue excision and biopsy	-	-	Granulation tissue with aural polyp excision and biopsy	-	Granulation tissue excision and biopsy
Biopsy results	Inflammatory aural polyp with dead bone	inflammatory aural polyp with dead bone	Inflammatory aural polyp	-	-	Inflammatory aural polyp with dead bone.	-	Granulation tissue
Surgical procedures	-	-	-	-	-	-	-	-
Diagnosis	Left SBO	Left SBO	Left SBO	Left SBO	Left SBO	Left SBO	Right SBO	Right SBO
Summary of clinical presentation, outcome, and management of MOE cases								

**Figure 5:**
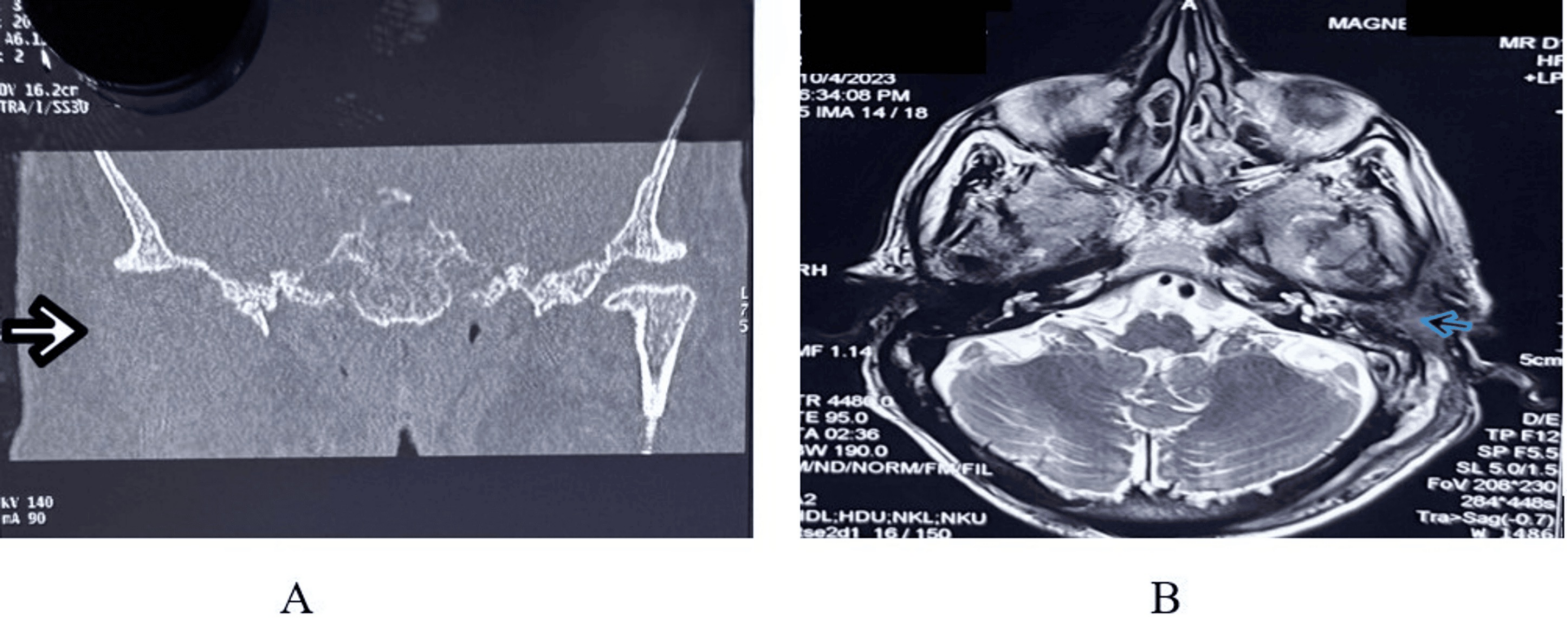
A: High-resolution computed tomography showing right TMJ septic arthritis. B: Magnetic resonance imaging of the brain A: High-resolution computed tomography showing right TMJ septic arthritis and anterior dislocation of the right TMJ. B: T2-weighted MRI showing hyperintense signal due to diffuse fluid accumulation filling the left middle ear cavity, mesotympanum, posterior epitympanum, and retrotympanic recess, with extension into the peri-ossicular region abutting the facial nerve canal. TMJ: temporomandibular joint

## Discussion

MOE has become manageable with the introduction of new antibiotics. However, the prognosis deteriorates with the onset of skull-base osteomyelitis or other complications. MOE is a potentially fatal infection affecting the lateral temporal bone. According to our study, the predominant symptoms of MOE include ear pain (nocturnal otalgia), ear discharge, and ear-blocking sensation [5].

As in our study, the data exhibited a mean of 65, ranging from a minimum of 50 to a maximum of 85. The increasing age has a significant impact on disease incidence. Several hypotheses have been proposed for the physiologic connection between advanced age and SBO, including decreased epithelial migration of the ear canal and microvascular disease inhibiting a proper immune response [2]. Advanced age has been linked to comorbidities, including diabetes, which is known to be associated with impaired immune response and microvascular disease [6].

The duration of DM and the patient’s serum glucose levels were considered to influence the prognosis of MOE [7]. Our study showed that an increase in blood glucose level (HbA1C) and irregular treatment had an impact on the disease course and severity.

In the available literature, men are more commonly affected than women [8]. Our study similarly observes a higher incidence of SBO in men as compared to women.

There are no studies signifying the incidence of affected sides in SBO. In our study, we observed that the left ear was affected more than the right ear, although the etiology of this could not be explained.

*Pseudomonas aeruginosa* has been identified as the predominant pathogen in 80-98% of skull base osteomyelitis cases [9]. In our case series, *Pseudomonas aeruginosa* was cultured in all patients. However, it is important to note that fungal pathogens, such as Aspergillus and Candida species, also play a significant role, particularly in immunocompromised patients, including those with hematologic malignancies or poorly controlled diabetes [10]. The prevalence of specific pathogens varies significantly across studies and patient populations, highlighting the need for individualized diagnostic approaches and targeted therapy. Additional studies reporting fungal infections as a cause of skull base osteomyelitis emphasize this variability.

CT is a fast and economical tool for confirming the diagnosis of SBO but has limited value in predicting outcomes [11]. Anatomical imaging enables the localization of the disease and assessment of its progression or resolution. Bone erosion can only be demonstrated on CT scans once bone demineralization has occurred [12]. However, HRCT of the temporal bone was taken in all our cases, through which the extent of the disease was assessed.

The facial nerve is the most commonly affected cranial nerve due to its proximity to the EAC [12]. In our study, three patients were affected by facial nerve palsy.

Over the past 15 years, oral ciprofloxacin has been the standard treatment for MOE, caused by *Pseudomonas aeruginosa*, due to which a gradual resistance to ciprofloxacin is developing. In a study over a 16-month period, 5 cases of SBO progressed, with the development of cranial nerve palsies in 4 cases, despite oral ciprofloxacin [8]. In our analysis, we noted that six cases with resistance to ciprofloxacin had an elevated risk of recurrences, advancing to facial nerve palsy, and developing septic arthritis of the TMJ. Therefore, patients with resistance to ciprofloxacin have a poor prognosis.

Otitis externa spreading to the TMJ is rare. In a study of two elderly patients with predisposing factors, it was found that a high index of suspicion and early aspiration of the joint enabled the diagnosis to be made and subsequent treatment to be instigated [13]. We reported three cases with early involvement of the TMJ joint, and a complete resolution was achieved. However, early involvement of the joint from SBO could be related to anatomical factors such as failure of closure of Huschke's foramen in early life; this leads to herniation of the TMJ into the anterior wall of the bony external ear canal [13].

A multidimensional approach, including surgical debridement, is particularly important when conservative treatment alone fails or when there is significant tissue involvement, highlighting the evolving understanding of SBO management in challenging patient populations [14]. Similarly in our study, four patients underwent surgical procedures, including incision and drainage of a left subperiosteal abscess, left mastoid exploration, and left aural polypectomy with EAC debridement. These interventions were necessary for patients with refractory or extensive SBO, where conservative treatment alone was insufficient. Surgical debridement, combined with antimicrobial therapy, was crucial in controlling infection and improving outcomes.

## Conclusions

In conclusion, our cases of SBO often present with otalgia, edema, and otorrhea. Physicians must be aware of the possibility that elderly, diabetic, and immunocompromised patients with persistent otalgia after refractory external otitis media may be suffering from SBO. Timely diagnosis and treatment significantly improve the prognosis of SBO. Computed tomography scans have been instrumental in diagnosing SBO. Our study revealed a noticeable tendency toward a higher occurrence on the left side in SBO cases. For this, further research is required to form a hypothesis. Poor prognosis is seen in patients with resistance to ciprofloxacin. Therefore, with early diagnosis, good control over the glycemic index, prolonged medical management, and local debridement, patients are likely to have a better prognosis.
